# Comparison of anterior knee laxity, stiffness, genu recurvatum, and general joint laxity in the late follicular phase and the ovulatory phase of the menstrual cycle

**DOI:** 10.1186/s12891-021-04767-8

**Published:** 2021-10-18

**Authors:** Mayuu Shagawa, Sae Maruyama, Chie Sekine, Hirotake Yokota, Ryo Hirabayashi, Arisa Hirata, Mizuki Yokoyama, Mutsuaki Edama

**Affiliations:** 1 Department of Physical Therapy, Niigata University of Health and Welfare, 1398 Shimami-cho, Kita-ku, Niigata, 950-3198 Japan; 2 Institute for Human Movement and Medical Sciences, Niigata University of Health and Welfare, 1398 Niigata, Shimami-cho 1398, Kita-ku, Niigata City, 950-3198 Japan

**Keywords:** Anterior cruciate ligament, Hormone, estradiol concentration, Hyper knee extension, Hyper joint mobility

## Abstract

**Background:**

One risk factor for anterior cruciate ligament (ACL) injury may be fluctuations in female hormones. This study examined variability in joint laxity, as a risk factor for ACL injury, during the menstrual cycle.

**Methods:**

Subjects were 15 female university students with regular menstrual cycles. We measured estradiol (E2) concentration, anterior knee laxity (AKL), stiffness, genu recurvatum (GR), and general joint laxity (GJL) during the late follicular and ovulatory phases. AKL was measured as anterior tibial displacement of the femur after application of 44-, 89-, and 133-N loads on the tibia. Stiffness was calculated as Δforce/Δdisplacement at loads of 44–89 N and between 89 and 133 N. GR was measured prone, with the base of the patella distal to the edge of the bed. The University of Tokyo joint laxity test was used to evaluate GJL.

**Results:**

E2 concentration was significantly higher in the ovulatory phase than in the late follicular phase (*p* = 0.018), AKL and stiffness did not differ significantly between phases, and GR and GJL were significantly higher in the ovulatory phase than in the late follicular phase (*p* = 0.011, 0.031).

**Conclusion:**

These findings suggest that E2 concentrations may affect GR and GJL during the menstrual cycle.

## Introduction

The incidence of anterior cruciate ligament (ACL) injury is higher in women than in men [1]. One reason for this difference is likely to be hormonal fluctuations [2]. The female menstrual cycle is regulated by the female hormones estrogen and progesterone [3], and is mainly classified into follicular, ovulatory, and luteal phases [4, 5]. The incidence of ACL injury during the menstrual cycle is not constant, with some reports indicating a higher incidence before ovulation than after ovulation [3], and a lower incidence during the luteal phase compared to the follicular and ovulatory phases [5]. Since the incidence of ACL injury differs between each phase of the menstrual cycle, fluctuations in female hormones may be involved in the development of ACL injury [4, 5].

The ACL possesses receptors for estrogen and progesterone [6], and in vitro studies of the ACL have shown that increasing the estradiol (E2) concentration, as a form of estrogen, causes a decrease in human ACL fibroblast proliferation and Type I procollagen synthesis [7]. On the other hand, progesterone decreases the effects of increased E2 concentration on ACL tissue metabolism [8]. Fluctuations in female hormones would thus affect tissue metabolism in the ACL, and ACL tissue metabolism may affect ACL extensibility, leading to changes in anterior knee laxity (AKL). Greater AKL is one risk factor for ACL injury [9, 10]. The relationship between female hormones and AKL during the menstrual cycle has been investigated in vivo using knee arthrometers such as the KT-1000 and KT-2000 (MED metric Corp, San Diego, CA, USA) [11–14]. Some reports have shown increases in AKL with increasing E2 concentration, but other similar studies have reported no significant changes in AKL during the menstrual cycle [13, 14], so changes AKL during menstrual cycle remain contentious.

In addition to AKL, genu recurvatum (GR) and greater general joint laxity (GJL) have also been reported to be associated with increased risk of ACL injury [15–17]. In a previous study that examined variations in GR and GJL during the menstrual cycle, GR was higher in the early luteal phase than in the early follicular phase [18]. In that study, Shultz et al. [18] measured GR in subjects in a supine position by voluntarily contracting the knee extensors. Since Changes in E2 concentration during the menstrual cycle could affect neuromuscular control function [19], observation of changes in peripheral tissues only, excluding central nervous systems, is more appropriate to measure GR passively. Clarification of the relationship between menstrual cycle and risk factors for ACL injury is important for establishing prevention and training methods for ACL injury.

One reason for the lack of consensus on variations in AKL during the menstrual cycle is the lack of uniformity in cycle classification among studies. In some studies, the menstrual cycle was classified only by the length of the cycle, and the timing of measurement was inconsistent among studies [13, 20]. In addition, individual differences exist in the magnitude and timing of female hormone fluctuations [21], so the relationship between hormone fluctuations and physical changes cannot be referred to based solely on the length of the cycle. Measurement of hormone concentrations therefore seems necessary to confirm cycle phases. In addition to hormone concentration measurement, it is possible to classify cycles more accurately using basal body temperature measurement and ovulation kits together.

The purpose of this study was therefore to examine changes in AKL, stiffness, GR, and GJL during the late follicular phase and ovulation phase of the menstrual cycle. We hypothesized that AKL, stiffness, GR, and GJL would increase during the ovulatory phase, when E2 concentrations are higher than in the late follicular phase.

## Methods

### Subjects

**Fig. 1:**
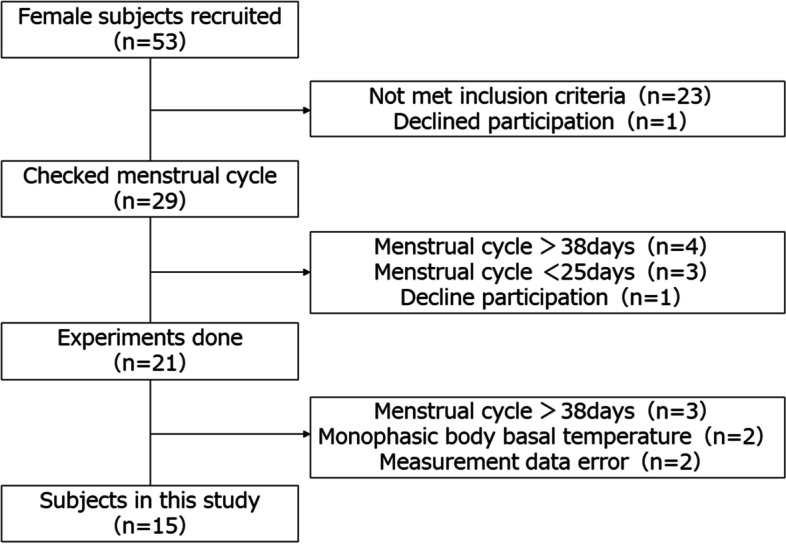
Subject selection process. Fifty-three female university students were surveyed, with 23 excluded after not meeting the inclusion criteria. For the remaining 29 students who agreed to participate in the study, their menstrual cycles were checked, and 7 were excluded because of cycle abnormalities. Of the 21 students who completed measurements, 2 with cycle abnormalities, 2 with monophasic basal body temperatures, and 2 with measurement errors were excluded. A final total of 15 female university students with biphasic basal body temperatures and normal menstruation were included

Fifty-six female university students were interviewed and given a questionnaire to determine if they met the study criteria. Inclusion criteria were as follows: 1) have a regular menstrual cycle with a length of 25–38 days [22]; 2) have a biphasic basal body temperature [23]; 3) have no history of damage to the knee joint, including osteochondral cartilage, ligament, tendon, joint capsule, or meniscus [18]; 4) have not used oral contraceptives or other hormonal agents within the past 6 months [18]; and 5) have no current exercise habit more than twice a week [24]. Fifteen subjects (mean age, 21 ± 0.3 years; height, 160.3 ± 5.6 cm; weight, 52.7 ± 10.6 kg; cycle length, 29 ± 2.8 days) met the inclusion criteria and agreed to participate in the study (Fig. 1). The study was performed in accordance with the Declaration of Helsinki after approval by the Ethics Committee of our institution (approval number: 17946). The study content was fully explained to the subjects, and written, informed consent was obtained from all subjects before they participated in the study.

### Confirmation of the menstrual cycle

The subjects were asked to measure their basal body temperature upon waking up every morning from 1 to 2 months before the start of the experiment. Basal body temperature was measured using a basal body thermometer (Citizen Electronic Thermometer CTEB503L; Citizen Systems Co., Tokyo, Japan). To estimate the day of ovulation, subjects were given an ovulation kit (Doctor’s Choice One Step Ovulation Test Clear; Beauty and Health Research, Inc., Torrance, CA, USA). A basal body temperature chart was prepared as a record sheet, and daily basal body temperature, menstrual period, and results of the ovulation kit were recorded. The transition to the high-temperature phase of basal body temperature coincides with or precedes ovulation [23]. We thus judged that basal body temperature shifted from the lower to the higher temperature phase and appeared biphasic when basal body temperature on the three consecutive days after the estimated day of ovulation was ≥0.2 °C higher than the average basal body temperature of the six consecutive days before the estimated day of ovulation [23].

### Timing of measurements

E2 concentration, AKL, stiffness, GR, and GJL were measured three times each during the late follicular phase and ovulatory phase, for a total of six times. The late follicular phase was measured during 3 days from the 2nd to 5th day after the end of menstruation, and the ovulatory phase was measured during 3 days from the 2nd to 5th day after the day on which the ovulation kit showed positive results. To account for diurnal variations, measurements were taken between 08:00 and 12:00 [12]. Temperature in the room was set to 20–25 °C.

### Measurement method

E2 concentration was measured using a saliva collection kit (SalivaBio A Salimetrics LLC Company, and the following points were strictly obeyed by subjects prior to saliva collection to avoid possible influence on E2 concentration: 1) prohibition of food intake within 60 min; 2) prohibition of alcohol intake within 12 h; 3) prohibition of sugary, acidic, or caffeinated drinks within 20 min; 4) prohibition of dairy products within 20 min; and 6) prohibition of saliva collection within 48 h after dental treatment. In addition, subjects were asked to rinse their mouths before the start of the experiment so that any food particles would not remain in the mouth, and saliva samples were taken > 10 min after rinsing the mouth, to prevent decreases in E2 concentration. Saliva was collected in the mouth for 1 min, then ejected into a saliva collection container (Cryovial; SAL) using a special straw (Siva Collection Aid; SAL). The saliva sample was immediately frozen in a freezer at less than − 80 °C after collection. After collecting all samples, analysis of E2 concentrations was entrusted to Funakoshi Corporation (Tokyo, Japan). E2 concentrations were determined using the High Sensitivity salivary 17β-Estradiol Enzyme Immunoassay Kit (SALIMETRICS), with samples thawed at room temperature, mixed by vortexing, centrifuged at 1500×*g* for 15 min, and analyzed by enzyme-linked immunosorbent assay. Dilutions were uniformly 1-fold dilution (undiluted solution).

**Fig. 2:**
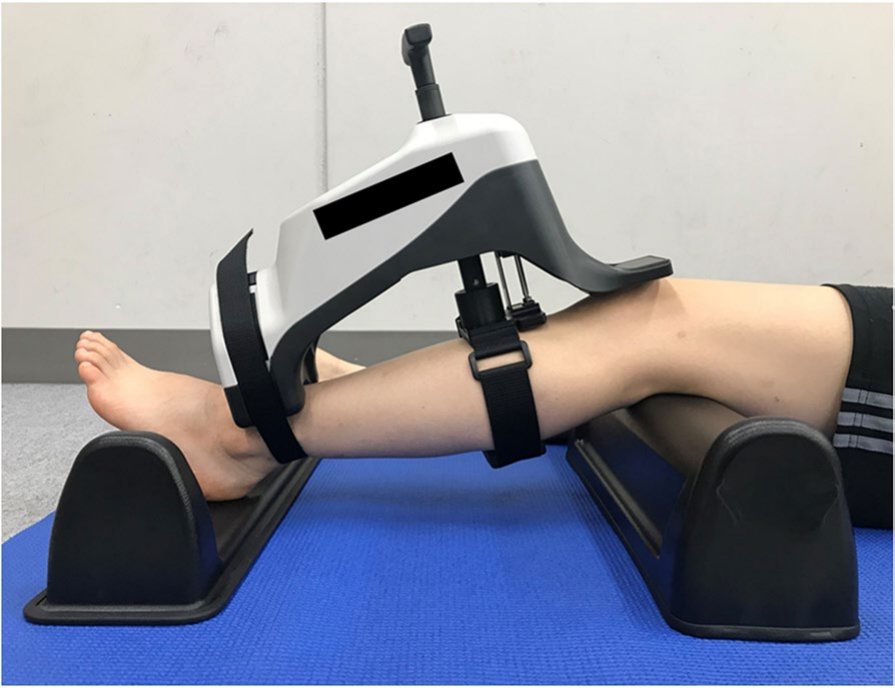
Position for measurement of anterior knee laxity. The subject was placed in a supine position, with the knee support placed on the distal posterior surface of the thigh, and the foot support placed under the foot to achieve approximately 30° of knee flexion. The position of the KS Measure was adjusted so that the area of patellar contact was centered on the knee and the area of ankle fixation was centered on the ankle. The lower leg was fixed with the traction belt and the ankle fixation belt

AKL was measured as anterior tibial displacement of the femur after application of 44-, 89-, and 133-N loads to the tibia, referring to a previous study [25]. The KS Measure (KS Measure KSM-100; Mark Electronics Co., Kanagawa, Japan) was used for measurement, following the measurement standards of Japan Sigmax Corporation (Tokyo, Japan). The subject was placed in a supine position, a knee rest was placed under the distal posterior surface of the thigh and a footrest was placed under the foot so that knee joint flexion was approximately 30° using a goniometer (Goniometer; Nishikawashinwa, Tokyo, Japan). The position of the KS Measure was adjusted so that the area of patellar contact was centered on the knee and the area of ankle contact was centered on the ankle. A traction belt was attached to the lower leg and a lower limb fixation belt was attached to the ankle. The subject was instructed to relax, and the measurement was made by pulling the load handle (Fig. 2). Five measurements were performed on the subject’s axle leg which opposite to the one kicking the ball, and the average of the three remaining measurements after excluding the maximum and minimum values was used as the measurement value. Knee joint angle measurement was performed by two examiners: one operating the load handle and one controlling the KS Measure panel. Stiffness was calculated as Δforce/Δdisplacement at loads between 44 and 89 N and between 89 and 133 N.

**Fig. 3:**
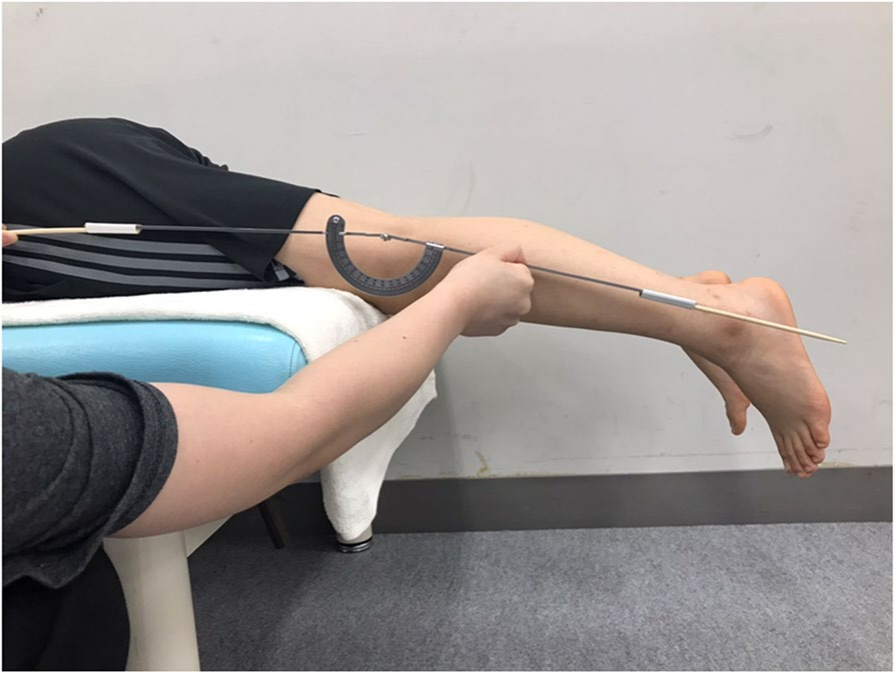
Position for measurement of genu recurvatum. The subject was placed in a prone position on the bed, with the base of the patella distal to the edge of the bed [26]. The hip joint was set at 0° of internal and external rotation. The basic axis was from the greater trochanter of the femur to the lateral femoral epicondyle, and the translation axis was from the head of the fibula to the lateral malleolus

GR was measured using a goniometer to evaluate the range of motion of knee extension. The subject was placed in a prone position on the bed, and the base of the patella was positioned distal to the edge of the bed [26]. The hip joint was set at 0° of internal and external rotation. The basic axis was from the greater trochanter of the femur to the lateral epicondyle of the femur, and the translation axis was from the head of the fibula to the lateral malleolus (Fig. 3). Measurements were taken three times, using the mean value as the measurement value. Two examiners, one to evaluate and one to manually restrain the pelvis to prevent lifting, performed the measurements.

**Fig. 4:**
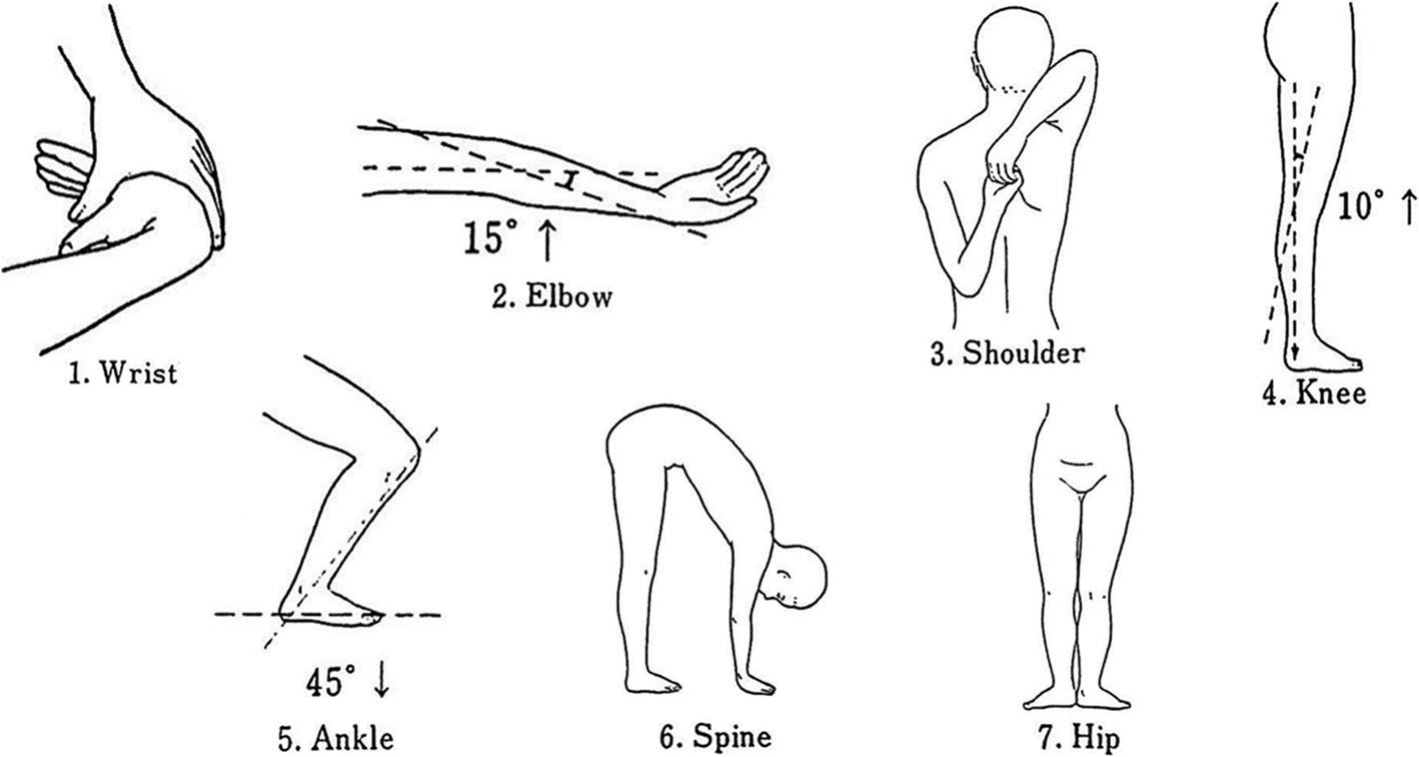
The University of Tokyo joint laxity test. This test evaluates joint laxity in the six major joints of the body and in the spine. A score is given when the condition for each parameter is met. Scores for Joints 1–5 (Wrist, Elbow, Shoulder, Knee, and Ankle, respectively) are 0.5 points for each side, and scores for Joints 6 and 7 (Spine and Hip) are 1 point. Maximum score is 7 points

The University of Tokyo joint laxity test was used for evaluation of GJL [27] (Fig. 4). One point was recorded for each component criterion that was met (0.5 points for each side), for a total score of 0–7. Components including a joint angle as a criterion were evaluated using a goniometer. The order of measurements was as follows: GJL was measured first, then AKL and GR were measured randomly. Finally, saliva samples were collected.

### Evaluation of intra-examiner reproducibility

The reproducibility of AKL and GR measurements was examined in 10 adult males (mean age, 21 ± 0.7 years; height, 173 ± 6.2 cm; weight, 68 ± 9.9 kg) with no history of orthopedic conditions and no pain in the lower limbs. Reproducibility was calculated using the intraclass correlation coefficient (ICC)(1, 3).

### Statistical analysis

Two-way repeated-measures analysis of variance was used for comparison of AKL and stiffness between the two phases. Corresponding t-test was used for comparison of E2 concentration, GR, and GJL between phases. The significance level was set at 5%. Statistical analyses were performed using SPSS version 24.0) (Tokyo, Japan).

## Results

The ICC (1,3) values for AKL were 0.871 (44 N), 0.827 (89 N), and 0.816 (133 N). The ICC (1,3) values for GR was 0.894. According to the criterion of Landis et al. [28], reproducibility is considered almost perfect for ICC values ≥0.81, so the reproducibility was considered sufficient. Data for E2 concentration, AKL, stiffness, GR, and GJL in the late follicular and ovulatory phases are shown in Table 1. E2 concentration was significantly higher in the ovulatory phase than in the late follicular phase (*p* = 0.018), AKL did not differ significantly between phases, and GR and GJL were significantly higher in the ovulatory phase than in the late follicular phase (*p* = 0.011, 0.031).

**Table 1:** Comparison of Estradiol concentration, Anterior Knee Laxity, Genu Recurvatum, and General Joint Laxity by two phases (late follicular and ovulatory)

	Late follicular phase	Ovulatory phase
E_2_ concentration [pg/mm]	0.95 ± 0.12	1.11 ± 0.28*
AKL [mm]		
44 N	3.35 ± 1.44	3.06 ± 1.34
89 N	5.66 ± 1.76	5.31 ± 1.82
133 N	7.42 ± 2.09	7.06 ± 2.12
Stiffness [N/m]		
44-89 N	21.61 ± 7.19	21.75 ± 6.30
89-133 N	27.83 ± 8.52	27.17 ± 7.47
GR		
Angle [°]	6.37 ± 2.57	7.32 ± 2.32*
GJL		
Score [points]	1.98 ± 1.35	2.21 ± 1.32*

Values are mean ± SD (*n* = 15)

*E2* estradiol, *AKL* anterior knee laxity, *GR* genu recurvatum, *GJL* general joint laxity

* *p* < 0.05 vs. late follicular phase

## Discussion

In this study, AKL and stiffness did not show significant differences between the late follicular and ovulatory phases, consistent with previous studies reporting that AKL does not fluctuate during the menstrual cycle [13, 14]. Some studies have reported no direct correlation between AKL and E2 concentration [11], and that fluctuations in E2 concentration may delay AKL by 3–4 days [29], suggesting that the timing of fluctuations in E2 concentration and AKL may not be synchronized. On the other hand, other studies have reported that AKL increased in the ovulatory phase compared to the early follicular phase [12, 20, 21]. The reason for such discrepancies between the present results and the findings of previous studies is presumably the difference in the timing of measurements: although most reports of increased AKL compared the early follicular phase with the ovulatory phase [5, 12, 20, 21], the present study compared the late follicular phase with the ovulatory phase. The late follicular phase is the period when E2 concentration gradually increases, but we avoided the early follicular phase because it may discomfort the subject to be in the prone position for measurement during the menstruation. However, E2 concentration was significantly higher in the ovulatory phase than in the late follicular phase, so we could examine the relationship between variations in E2 concentration and AKL in the two periods. Therefore, it is possible that AKL does not fluctuate between the late follicular phase and the ovulatory phase.

GR was significantly increased in the ovulatory phase compared with the late follicular phase. In this study, GR was measured as the range of motion of knee joint extension. Stability of the posterior knee joint is not only restricted by bony structures, but also by the soft tissues surrounding the knee joint, such as the posterior capsular ligament, ACL, posterior cruciate ligament, oblique popliteal ligament, hamstrings, and gastrocnemius muscle [30]. A previous study confirmed that E2 receptors present in skeletal muscle [31]. The present results suggest that E2 concentration may affect skeletal muscle, and that the knee in extension and GJL may change during the menstrual cycle. However, the clinical significance of the measured difference in GR of only 1° between the two phases has not been investigated and is unknown. There may be some significance to the change from 9° to 10°, because ACL injury is more common in those with GR more than 10° [32]. The clinical significance in GR needs to be investigated in the future.

GJL was significantly higher in the ovulatory phase than in the late follicular phase, suggesting that the soft tissues around the joints may have changed. Shultz et al. [18] reported that GJL changed with menstrual cycle, suggesting that hormonal fluctuations may affect joint laxity.

A key limitation of this study was that although both GR and GJL assessed joint laxity, we were unable to determine which specific tissues both inside and outside the joint were affected in this study. Examination of the relationship between E2 and factors that increase GR and GJL will be necessary in the future. In addition, only 25% of all recruited subjects were included in the data analysis which is potentially limit the generalizability of the data. The most of subjects who did not inclusion criteria by their history of damage to the knee joint, including osteochondral cartilage, tendon, joint capsule, or meniscus. The number of subjects who excluded by using oral contraceptives is only two. Further, reports have suggested that relaxin, progesterone, and testosterone are associated with ACL injury [33, 34]. Other female hormones may therefore affect AKL, stiffness, GR, and GJL, and the relationships with other female hormones should therefore be considered.

## Conclusions

Among female university students with regular menstrual cycles, we found no significant differences in AKL or stiffness between the late follicular and ovulatory phases. GR and GJL were higher in the ovulatory phase than in the late follicular phase. Variations in E2 concentration during the menstrual cycle may thus affect variations in GR and GJL.

## Data Availability

The datasets generated and/or analysed during the current study are not publicly available due to limitations of ethical approval involving the patient data and anonymity but are available from the corresponding author on reasonable request.
